# The Hildebrand Solubility Parameters of Ionic Liquids—Part 2

**DOI:** 10.3390/ijms12063553

**Published:** 2011-06-03

**Authors:** Andrzej Marciniak

**Affiliations:** Department of Physical Chemistry, Faculty of Chemistry, Warsaw University of Technology, Noakowskiego 3, 00-664 Warsaw, Poland; E-Mail: a.marciniak@ch.pw.edu.pl; Tel.: +48-222-345-816; Fax: +48-226-282-741

**Keywords:** ionic liquid, Hildebrand solubility parameter, enthalpy of vaporization

## Abstract

The Hildebrand solubility parameters have been calculated for eight ionic liquids. Retention data from the inverse gas chromatography measurements of the activity coefficients at infinite dilution were used for the calculation. From the solubility parameters, the enthalpies of vaporization of ionic liquids were estimated. Results are compared with solubility parameters estimated by different methods.

## 1. Introduction

Ionic liquids (ILs) are a relatively new class of salts with a melting temperature below 373.15 K. In general, ILs are composed of organic cations with either inorganic or organic anions. Ionic liquids have unique properties, namely, a wide liquid range, stability at high temperatures and negligible vapor pressure. Because of the last mentioned property, the inverse gas chromatography (IGC) is a suitable method for measuring thermodynamic properties of pure substances and their mixtures [1]. From the IGC measurements, the activity coefficients at infinite dilution, Flory-Huggins interaction parameters as well as the Hildebrand solubility parameters can be determined. By this method the solubility parameters were determined previously for different ionic liquids [2–6].

The Hildebrand solubility parameters have numerous applications including gas-liquid solubility, solvent extraction and many others as described in detail in the literature [7,8]. The solubility parameter is the square root of the cohesive energy density, which is defined as the ratio of the energy of vaporization, Δ_vap_*U*, to the molar volume, *υ*:

(1) $$\delta =\sqrt{\frac{\Delta_{\text{vap}}U}{\upsilon }}=\sqrt{\frac{\Delta_{\text{vap}}H-\text{R}T}{\upsilon }}$$

Because ILs have negligible vapor pressure, experimental measurements of their energy of vaporization are difficult. For this reason, experimental data of Δ_vap_*U* are unavailable. Alternative methods have been considered for estimation of the solubility parameters of ionic liquids: From melting temperatures of ILs [9], from intrinsic viscosity measurements [10], from the activation energy of viscosity [11,12], from surface tension measurements [13], from Kamlet-Taft equation [14], using non random hydrogen bonding (NRHB) and PC-SAFT models [15], from lattice energy density [16].

This paper provides information on the Hildebrand solubility parameters determined for eight ionic liquids as a function of temperature and the enthalpies of vaporization calculated from the values of the solubility parameters. The solubility parameters were calculated using the experimental data from the activity coefficients at infinite dilution measurements. The list of investigated ionic liquids is shown in Table 1. The values of the activity coefficients at infinite dilution for the investigated ionic liquids were published earlier [17–24].

## 2. Results and Discussion

The Hildebrand solubility parameters were calculated for the ionic liquids presented (with abbreviations and structures) in Table 1. The results are presented in Table 2. For ionic liquids based on [FAP]^−^ and [NTf_2_]^−^ anions with the same cation, [*N*-C_3_OHPY]^+^, the solubility parameter is higher for IL with [NTf_2_]^−^ anion. Estimated enthalpy of vaporization is higher for [*N*-C_3_OHPY][FAP] than for [*N*-C_3_OHPY][NTf_2_], the higher molar mass and more complex structure of [FAP]^−^ anion causes higher enthalpy of vaporization. For ionic liquids [bmPIP][SCN] and [bmPIP][NTf_2_] the solubility parameter as well as the enthalpy of vaporization is higher for ionic liquid with [SCN]^−^ anion. In this case the structure of [SCN]^−^ anion is much simpler than for [NTf_2_]^−^ and the molar mass is lower, but very strong interaction of thiocyanate group increases the enthalpy of vaporization. With an increase of the alkyl chain in the cation structure of an ionic liquid the solubility parameter decreases. Due to increase of molar mass and alkyl chain length the enthalpy of vaporization also increases. This is typical behavior observed with increasing of alkyl chain length for example in linear alkanes or alkylbenzenes. This effect is visible in two pairs of ionic liquids, namely [emim][TCB]^−^[dmim][TCB] and [pmPIP][NTf_2_]^−^[bmPIP][NTf_2_].

Table 3 presents comparison of the Hildebrand solubility parameters determined by different methods for selected ionic liquids based on [NTf_2_]^−^ anion. Camper *et al*. presents different values of *δ* for ionic liquid [emim][NTf_2_] estimated from the IL melting point [9] and from lattice energy density [16]. These values differ about 2.4 times and are inconsistent with *δ* obtained by other methods. Solubility parameters determined from enthalpy of vaporization are in good agreement with values of *δ* obtained by IGC for [emim][NTf_2_] and [hmim][NTf_2_] and with values of *δ* estimated from surface tension for [bmim][NTf_2_] and [bmPYR][NTf_2_]. Kilaru *et al*. estimated solubility parameters from activation energy of viscosity using the equation presented below [11]:

(2) $$\delta ={\left[\frac{K_{\text{v}}RT}{\upsilon }\text{ln}\left(\frac{10^{-9}\mu \upsilon }{{hN}_{\text{A}}}\right)\right]}^{0.5}$$

where: *μ* is the dynamic viscosity of IL (in units of mPa·s), *υ* is the molar volume (in units of cm^3^·mol^−1^), *h* is Planck constant (in units of J·s), *N*_A_ is Avogadro constant (in units of mol^−1^), and *K*_v_ is a proportionality constant. They calculated *K*_v_ value of 7.8 for ILs based on [NTf_2_]^−^ anion from solubility parameters determined from intrinsic viscosity [10]. Consequently the solubility parameters estimated from Equation 2 are consistent with those estimated from intrinsic viscosity. In this work *K*_v_ value of 5.23 was obtained from the solubility parameters determined from experimental enthalpy of vaporization (the procedure is described in Supporting Information). Based on this value the solubility parameters were determined for [*N*-C_3_OHPY][NTf_2_], [pmPIP][NTf_2_] and [bmPIP][NTf_2_] ionic liquids for which the molar volumes and viscosities were determined (see Table 3S). Results are presented in Table 4. The differences in results are in the range from 3 to 10%.

## 3. Calculation of Solubility Parameters

### 3.1. Experimental Procedure

On the basis of the experimental data from the activity coefficients at infinite dilution measurements, the Hildebrand solubility parameters have been calculated using the equations presented below. The activity coefficients at infinite dilution for all investigated ionic liquids were measured using inverse gas chromatography. Detailed descriptions of materials, apparatus and methods used in each experiment are presented in the relevant papers [17–24].

### 3.2. Theoretical Basis

Retention data were used for the calculation of Hildebrand solubility parameters, *δ*_2_. According to the Flory-Huggins theory the interaction parameter at infinite dilution can be determined using the following expression:

(3) $$\chi_{12}^{\infty }=\text{ln}\left(\frac{273.15\text{R}}{P_{1}^{*}V_{g}M_{1}}\right)-\frac{P_{1}^{*}(B_{11}-V_{1}^{*})}{\text{R}T}+\text{ln}\left(\frac{\rho_{1}}{\rho_{2}}\right)-\left(1-\frac{V_{1}^{*}}{V_{2}^{*}}\right)$$

where R denotes the gas constant, *T* the temperature, *P*_1_^*^ the saturated vapor pressure of the solute at temperature *T*, *B*_11_ the second virial coefficient of pure solute, *V*_1_^*^ and *V*_2_^*^ the molar volume of the solute and solvent respectively, *M*_1_ the molar mass of solute, *ρ*_1_ and *ρ*_2_ density of solute and solvent respectively, *V*_g_ specific retention volume which is given by:

(4) $$V_{g}=\frac{273.15V_{\text{N}}}{{Tm}_{2}}$$

where *m*_2_ denotes the mass of the solvent on the column packing and *V*_N_ the net retention volume of the solute given by:

(5) $$V_{\text{N}}=J_{2}^{3}U_{\text{o}}(t_{\text{R}}-t_{\text{G}})$$

where *t*_R_ and *t*_G_ are the retention times for the solute and an unretained gas, respectively, *U*_o_ is the column outlet flow rate, *J*_2_^3^ the pressure correction term given by:

(6) $$J_{2}^{3}=\frac{2}{3}\frac{{\left(P_{\text{i}}/P_{\text{o}}\right)}^{3}-1}{{\left(P_{\text{i}}/P_{\text{o}}\right)}^{2}-1}$$

where *P*_i_ and *P*_o_ denote the inlet and the outlet pressure, respectively. The column outlet flow rate corrected for the vapor pressure of water *U*_o_ is given by:

(7) $$U_{o}=U\left(1-\frac{P_{w}}{P_{o}}\right)\frac{T}{T_{f}}$$

where *T_f_* is the temperature at the column outlet, *P_w_* is the vapor pressure of water at *T_f_* and *U* is the flow rate measured with the flow meter. The interaction parameter *χ*_12_^∞^ may be expressed as a function of *δ*_1_ and *δ*_2_ which denote the solubility parameters of the solute and of the solvent, respectively, by:

(8) $$\chi_{12}^{\infty }=\frac{V_{1}^{*}{\left(\delta_{1}-\delta_{2}\right)}^{2}}{\text{R}T}$$

Equation 8 can be rewritten as:

(9) $$\left(\frac{\delta_{1}^{2}}{\text{R}T}-\frac{\chi_{12}^{\infty }}{V_{1}^{*}}\right)=\left(\frac{2\delta_{2}}{\text{R}T}\right)\delta_{1}-\frac{\delta_{2}^{2}}{\text{R}T}$$

The solubility parameters *δ*_1_ of the solutes were calculated using following equation:

(10) $$\delta^{2}=\frac{\Delta_{\text{vap}}H-\text{R}T}{\upsilon }$$

where Δ_vap_*H* denotes enthalpy of vaporization and *υ* the molar volume. The thermophysical properties required in calculations were calculated using equations and constants taken from the literature [30].

Values of *χ*_12_^∞^ were determined from Equation 2 and are presented in Table 1S. If the left side of Equation 9 is plotted against *δ*_1_, a straight line having a slope of 2*δ*_2_/R*T* and an intercept of −*δ*_2_^2^ /R*T* is obtained. The solubility parameter of the solvent *δ*_2_ (ionic liquid) can be calculated from the slope. Example of calculations is presented in the Supporting Information. Hildebrand solubility parameters of the investigated ionic liquids and the estimated enthalpies of vaporization calculated using Equation 10 are listed in Table 2.

## 4. Conclusions

The Hildebrand solubility parameters estimated by different methods are divergent. The most reliable results are from the experiment especially from the enthalpies of vaporization. As presented in Table 3, solubility parameters calculated from enthalpies of vaporization and determined by IGC are in good consistency for [emim][NTf_2_] and [hmim][NTf_2_] ionic liquids. Therefore, the inverse gas chromatography is an appropriate method to determine Hildebrand solubility parameters of ionic liquids. While the ionic liquids have negligible vapor pressure, experimental measurements of their enthalpy of vaporization are difficult; therefore, this property can be estimated from the solubility parameters.

## Figures and Tables

**Table 1:** Abbreviations, names, sources, purities and structures of investigated ionic liquids.

Abbreviation, Name, Source, Purity	Structure	Reference
abbreviation: [*N*-C_3_OHPY][FAP] name: 1-(3-hydroxypropyl)pyridinium trifluorotris(perfluoroethyl)phosphate source: MERCK purity > 0.999 mass fraction water content < 100 ppm halide content < 100 ppm	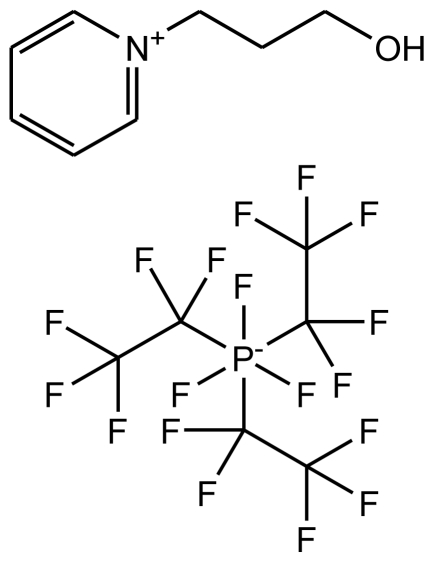	[17]
abbreviation: [*N*-C_3_OHPY][NTf_2_] name: 1-(3-hydroxypropyl)pyridinium bis(trifluoromethylsulfonyl)-amide source: MERCK purity > 0.999 mass fraction water content < 100 ppm halide content < 100 ppm	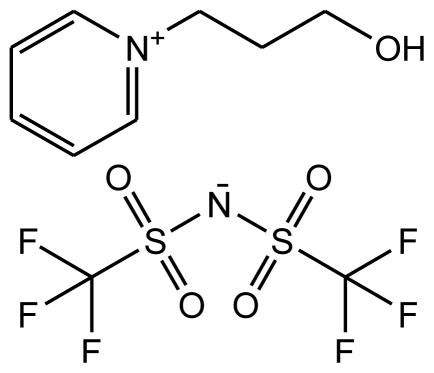	[18]
abbreviation: [emim][TCB] name: 1-ethyl-3-methylimidazolium tetracyanoborate source: MERCK purity > 0.99 mass fraction water content < 200 ppm halide content < 100 ppm	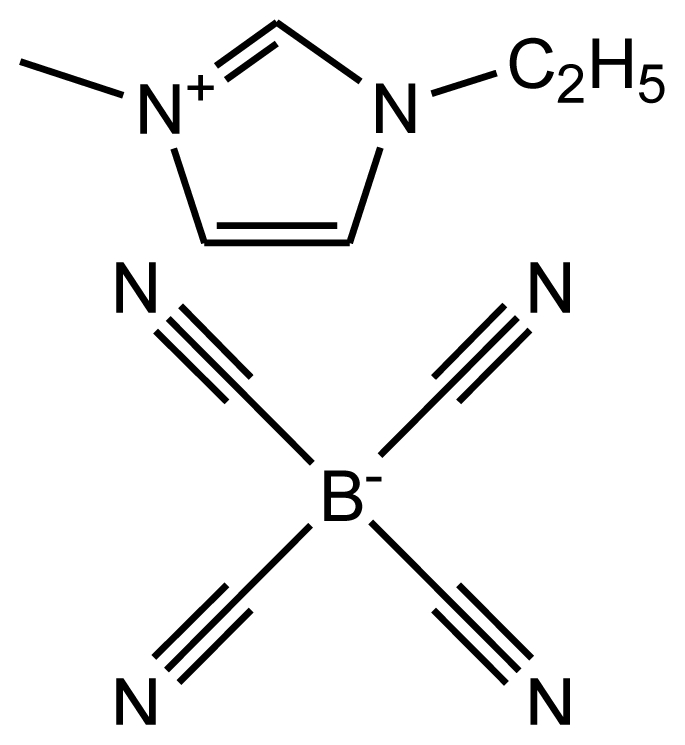	[19]
abbreviation: [dmim][TCB] name: 1-decyl-3-methylimidazolium tetracyanoborate source: MERCK purity > 0.9996 mass fraction water content: < 100 ppm halide content < 100 ppm	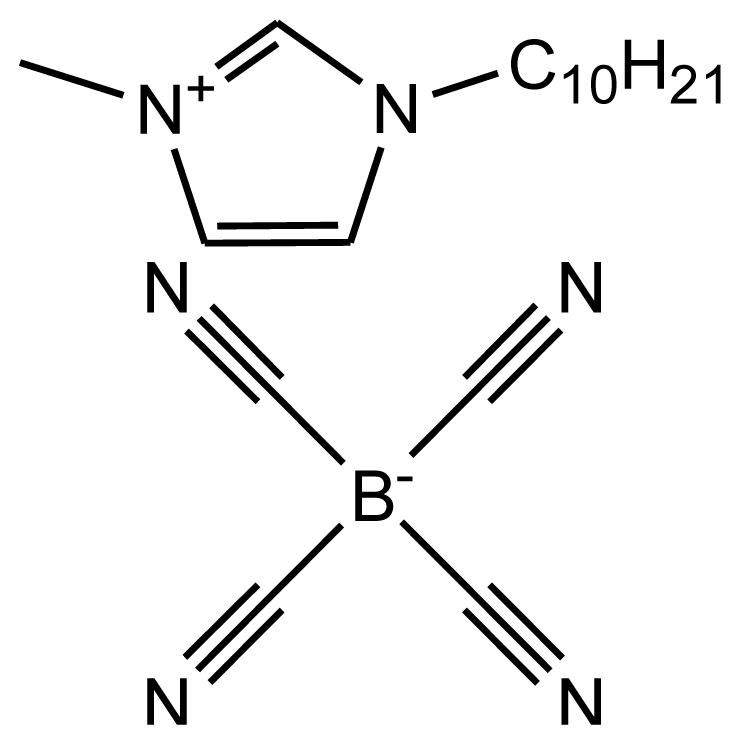	[20]
abbreviation: [bmPIP][SCN] name: 1-butyl-1-methylpiperidinium thiocyanate source: IoLiTec purity > 0.98 mass fraction water content: < 100 ppm halide content < 100 ppm	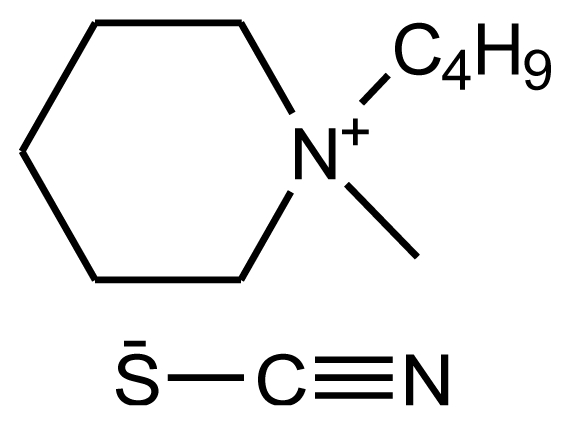	[21]
abbreviation: [pmPIP][NTf_2_] name: 1-propyl-1-methylpiperidinium bis(trifluoromethylsulfonyl)-amide source: IoLiTec purity > 0.99 mass fraction water content: < 100 ppm halide content < 100 ppm	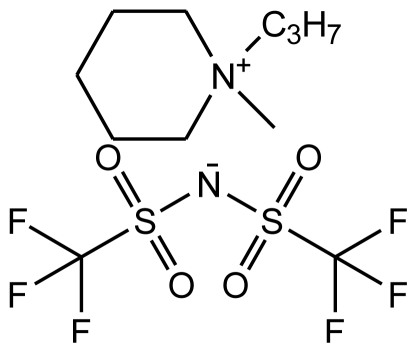	[22]
abbreviation: [bmPIP][NTf_2_] name: 1-butyl-1-methylpiperidinium bis(trifluoromethylsulfonyl)-amide source: IoLiTec purity > 0.99 mass fraction water content: < 250 ppm halide content < 100 ppm	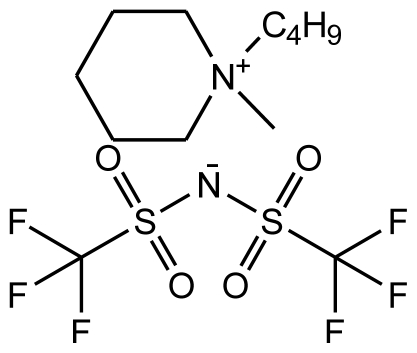	[23]
abbreviation: [OiQuin][NTf_2_] name: *N*-octyl-isoquinolinium bis(trifluoromethylsulfonyl)-amide source: synthesized purity > 0.99 mass fraction water content: < 180 ppm halide content < 100 ppm	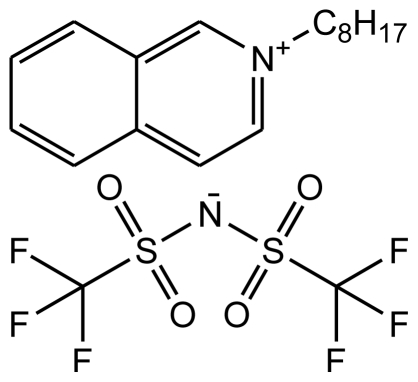	[24]

**Table 2:** Hildebrand solubility parameters, *δ*_2_ and standard enthalpies of vaporization for the investigated ionic liquids.

Ionic Liquid	*T*/K	*δ*_2_/MPa^0.5^	Δ_vap_*H*/kJ·mol^−1^
[*N*-C_3_OHPY][FAP]	298.15	25.0^a^	212.3
	308.15	24.7	209.6
	318.15	24.5	206.6
	328.15	24.2	203.3
	338.15	23.9	199.6
	348.15	23.6	196.2
	358.15	23.3	192.1

[*N*-C_3_OHPY][NTf_2_]	298.15	26.0^a^	186.1
	318.15	25.6	182.0
	328.15	25.3	179.5
	338.15	25.1	176.9
	348.15	24.8	174.2
	358.15	24.5	171.2

[emim][TCB]	298.15	25.9	149.5
	308.15	25.7	149.0
	318.15	25.5	147.9
	328.15	25.3	146.8
	338.15	25.1	145.6
	348.15	24.9	144.4
	358.15	24.6	142.6

[dmim][TCB]	298.15	24.0^a^	205.6
	328.15	23.6	201.9
	338.15	23.3	199.4
	348.15	23.1	197.1
	358.15	22.8	194.2
	368.15	22.5	190.5

[bmPIP][SCN]	298.15	30.7^a^	198.9
	318.15	30.1	193.4
	328.15	29.8	190.4
	338.15	29.5	187.2
	348.15	29.1	183.9
	358.15	28.8	180.5

[pmPIP][NTf_2_]	298.15	23.8^b^	172.4
	308.15	23.6	170.9
	318.15	23.3	167.9
	328.15	23.2	166.5
	338.15	22.9	164.2
	348.15	22.7	162.6
	358.15	22.5	160.7

[bmPIP][NTf_2_]	298.15	23.4^b^	175.1
	308.15	23.2	173.4
	318.15	23.0	171.7
	328.15	22.8	169.7
	338.15	22.6	168.0
	348.15	22.4	166.4
	358.15	22.2	164.6

[OiQuin][NTf_2_]	298.15	22.5^b^	201.3
	328.15	21.9	195.5
	338.15	21.7	193.2
	348.15	21.6	192.1
	358.15	21.4	189.7
	368.15	21.2	187.6

a Extrapolated values calculated using polynomial regression;

b Extrapolated values calculated using linear regression.

**Table 3:** Hildebrand solubility parameters, *δ*_2_ determined by different methods for selected ionic liquids based on [NTf_2_]^−^ anion at *T* = 298.15 K.

Ionic Liquid	*δ*_2_/MPa^0.5^	Method, Reference
[emim][NTf_2_]	16.2	melting temperature [9]
	19.3	activation energy of viscosity [12]
	21.3^b^	enthalpy of vaporization (Δ_vap_*H*_298.15_/kJ·mol^−1^ = 120.6) [25]
	22.3	IGC [4]
	22.6^b^	enthalpy of vaporization (Δ_vap_*H*_298.15_/kJ·mol^−1^ = 134) [26]
	22.7^b^	enthalpy of vaporization (Δ_vap_*H*_298.15_/kJ·mol^−1^ = 136) [27]
	27.5^a^	activation energy of viscosity [11]
	27.6	intrinsic viscosity [10]
	38.4	lattice energy density [16]

[bmim][NTf_2_]	19.8^b^	enthalpy of vaporization (Δ_vap_*H*_298.15_/kJ·mol^−1^ = 118.5) [25]
	20.9	activation energy of viscosity [12]
	21.2^b^	enthalpy of vaporization (Δ_vap_*H*_298.15_/kJ·mol^−1^ = 134) [26]
	21.3	surface tension [13]
	22.9^b^	enthalpy of vaporization (Δ_vap_*H*_298.15_/kJ·mol^−1^ = 155) [27]
	25.5	Kamlet-Taft Equation [14]
	26.5^a^	activation energy of viscosity [11]
	26.7	intrinsic viscosity [10]

[hmim][NTf_2_]	19.0^b^	enthalpy of vaporization (Δ_vap_*H*_298.15_/kJ·mol^−1^ = 124.1) [25]
	19.5	activation energy of viscosity [12]
	20.3	IGC [2]
	20.5^b^	enthalpy of vaporization (Δ_vap_*H*_298.15_/kJ·mol^−1^ = 139) [26]
	22.9^b^	enthalpy of vaporization (Δ_vap_*H*_298.15_/kJ·mol^−1^ = 173) [27]
	25.2^a^	activation energy of viscosity [11]
	25.6	intrinsic viscosity [10]

[omim][NTf_2_]	18.9^b^	enthalpy of vaporization (Δ_vap_*H*_298.15_/kJ·mol^−1^ = 132.3) [25]
	20.2^b^	enthalpy of vaporization (Δ_vap_*H*_298.15_/kJ·mol^−1^ = 149) [28]
	20.2^b^	enthalpy of vaporization (Δ_vap_*H*_298.15_/kJ·mol^−1^ = 149) [26]
	23.0^b^	enthalpy of vaporization (Δ_vap_*H*_298.15_/kJ·mol^−1^ = 192) [27]
	25.0	intrinsic viscosity [10]

[bmPY][NTf_2_]	20.6	IGC [2]
	21.2	activation energy of viscosity [12]

[bmPYR][NTf_2_]	21.1	from surface tension [13]
	22.2^b^	enthalpy of vaporization (Δ_vap_*H*_298.15_/kJ·mol^−1^ = 152) [29]

[*N*-C_3_OHPY][NTf_2_]	25.6^c^	IGC [this work]
	23.0^c^	activation energy of viscosity [this work]

[pmPIP][NTf_2_]	23.6^c^	IGC [this work]
	23.5^c^	NRHB [15]
	23.4^c^	PC-SAFT [15]
	22.2^c^	activation energy of viscosity [this work]

[bmPIP][NTf_2_]	23.2^c^	IGC [this work]
	21.8^c^	activation energy of viscosity [this work]

a at *T* = 303.15 K;

b calculated from experimental value of Δ_vap_*H*_298.15_;

c at *T* = 308.15 K.

**Table 4:** Hildebrand solubility parameters, *δ*_2_ determined by different methods for [*N*-C_3_OHPY][NTf_2_], [pmPIP][NTf_2_] and [bmPIP][NTf_2_] ionic liquids.

Ionic Liquid	*T*/K	IGC	Activation Energy of Viscosity
[*N*-C_3_OHPY][NTf_2_]	308.15	25.6	23.0
	318.15	25.3	22.8
	328.15	25.1	22.7
	338.15	24.8	22.6
	348.15	24.5	22.6

[pmPIP][NTf_2_]	308.15	23.6	22.2
	318.15	23.3	22.0
	328.15	23.2	21.9
	338.15	22.9	21.8
	348.15	22.7	21.8

[bmPIP][NTf_2_]	308.15	23.2	21.8
	318.15	23.0	21.6
	328.15	22.8	21.5
	338.15	22.6	21.4
	348.15	22.4	21.3

**Figure S1:**
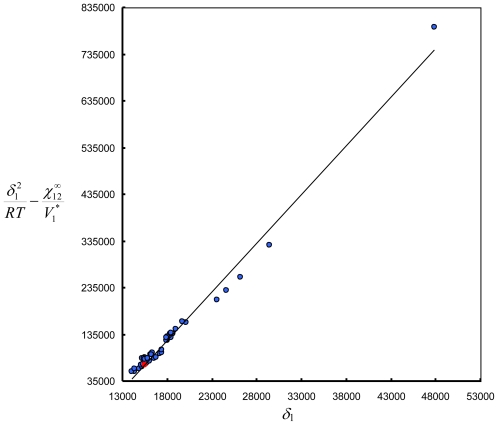
An example of the determination of solubility parameter *δ*_2_. Plot of $\frac{\delta_{1}^{2}}{RT}-\frac{\chi_{12}^{\infty }}{V_{1}^{*}}$ *versus δ*_1_ according to the Equation 7 for ionic liquid [emim][TCB] at *T* = 298.15 K. ( 
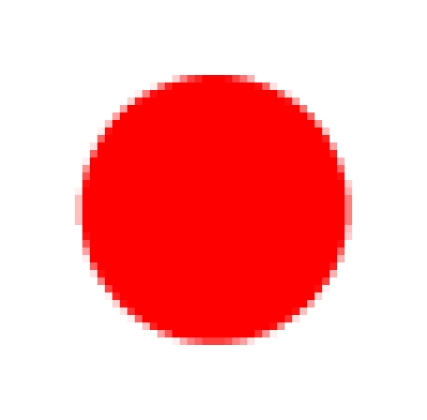
) *n*-octane, ( 
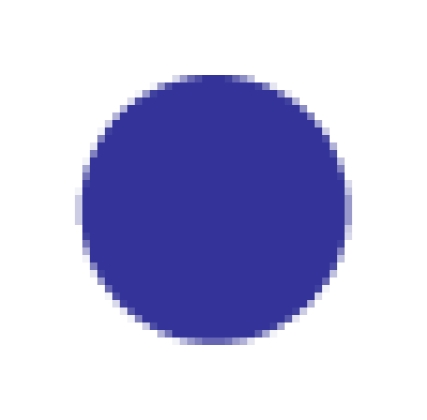
) rest of solutes.

## Appendix Electronic Supporting Information

Table S1, interaction parameters, *χ*_12_^∞^ Example of calculation of the solubility parameter. Calculation of the *K*_v_ constant from Equation 2; Table S2, data used in calculation of *K*_v_ constant; Table S3, densities and viscosities for [*N*-C_3_OHPY][NTf_2_], [pmPIP][NTf_2_] and [bmPIP][NTf_2_] ionic liquids.

**Table S1:** Interaction parameters, *χ* _12_^∞^.

*χ*_12_^∞^						
**[*****N*****-C****_3_****OHPY][FAP]**						

***T*****/K**	***n*****-pentane**	***n*****-hexane**	***n*****-heptane**	***n*****-octane**	***n*****-nonane**	***n*****-decane**
308.15	3.62	3.96	4.32	4.68	5.04	5.42
318.15	3.50	3.83	4.17	4.52	4.86	5.24
328.15	3.39	3.71	4.04	4.38	4.72	5.08
338.15	3.27	3.58	3.91	4.24	4.57	4.93
348.15	3.18	3.48	3.78	4.11	4.44	4.78
358.15	3.09	3.37	3.67	3.99	4.30	4.64
***T*****/K**	**cyclopentane**	**cyclohexane**	**cycloheptane**	**cyclooctane**	**1-pentene**	**1-hexene**
308.15	3.19	3.55	3.81	4.08	2.75	3.07
318.15	3.08	3.42	3.67	3.93	2.66	2.97
328.15	2.97	3.29	3.54	3.80	2.58	2.88
338.15	2.86	3.17	3.42	3.67	2.50	2.80
348.15	2.75	3.05	3.30	3.55	2.43	2.72
358.15	2.67	2.94	3.19	3.43	2.36	2.64
***T*****/K**	**1-heptene**	**1-octene**	**1-hexyne**	**1-heptyne**	**1-octyne**	**benzene**
308.15	3.40	3.79	2.01	2.33	2.69	0.482
318.15	3.31	3.68	1.96	2.28	2.63	0.495
328.15	3.22	3.58	1.92	2.23	2.57	0.505
338.15	3.14	3.48	1.87	2.18	2.51	0.519
348.15	3.06	3.38	1.83	2.13	2.45	0.528
358.15	2.97	3.29	1.79	2.08	2.40	0.537
***T*****/K**	**toluene**	**ethylbenzene**	***o*****-xylene**	***m*****-xylene**	***p*****-xylene**	**methanol**
308.15	0.732	1.12	0.944	1.01	1.06	1.01
318.15	0.743	1.12	0.950	1.02	1.07	0.979
328.15	0.751	1.12	0.955	1.03	1.07	0.944
338.15	0.761	1.12	0.961	1.04	1.08	0.913
348.15	0.769	1.12	0.966	1.04	1.08	0.882
358.15	0.777	1.11	0.971	1.05	1.09	0.855
***T*****/K**	**ethanol**	**1-propanol**	**1-butanol**	**water**	**thiophene**	**tetrahydrofuran**
308.15	0.873	1.01	1.20	2.95	0.557	−0.967
318.15	0.832	0.965	1.14	2.85	0.564	−0.859
328.15	0.796	0.916	1.08	2.77	0.572	−0.776
338.15	0.761	0.878	1.03	2.69	0.578	−0.683
348.15	0.730	0.836	0.974	2.61	0.585	−0.601
358.15	0.699	0.803	0.926	2.54	0.591	−0.529
***T*****/K**	**methyl*****tert-*****butyl ether**	**diethyl ether**	**di-*****n*****-propyl ether**	**di-*****n*****-butyl ether**	**2-pentanone**	**3-pentanone**
308.15	−0.0104	0.168	1.29	2.14		
318.15	0.083	0.245	1.32	2.15	−1.09	−0.988
328.15	0.170	0.324	1.36	2.15	−1.01	−0.907
338.15	0.262	0.390	1.39	2.16	−0.933	−0.831
348.15	0.335	0.450	1.43	2.16	−0.860	−0.759
358.15	0.414	0.504	1.45	2.16	−0.792	−0.694
***T*****/K**	**acetone**					
308.15	−1.66					
318.15	−1.56					
328.15	−1.47					
338.15	−1.38					
348.15	−1.30					
358.15	−1.23					

**[*****N*****-C****_3_****OHPY][NTf****_2_****]**						

***T*****/K**	***n*****-pentane**	***n*****-hexane**	**3-methylpentane**	**2,2-dimethylbutane**	***n*****-heptane**	***n*****-octane**
318.15	3.67	4.04	3.90	3.81	4.45	4.86
328.15	3.59	3.94	3.80	3.71	4.34	4.73
338.15	3.50	3.84	3.70	3.61	4.22	4.61
348.15	3.43	3.76	3.62	3.53	4.13	4.50
358.15	3.35	3.67	3.54	3.44	4.03	4.40
***T*****/K**	**2,2,4-trimethylpentane**	***n*****-nonane**	***n*****-decane**	**cyclopentane**	**cyclohexane**	**methylcyclohe xane**
318.15	4.40	5.27	5.70	3.08	3.45	3.78
328.15	4.30	5.13	5.54	2.99	3.35	3.68
338.15	4.20	5.00	5.41	2.91	3.26	3.58
348.15	4.11	4.89	5.29	2.84	3.18	3.49
358.15	4.03	4.77	5.16	2.78	3.10	3.41
***T*****/K**	**cycloheptane**	**cyclooctane**	**1-pentene**	**1-hexene**	**cyclohexene**	**1-heptene**
318.15	3.73	4.03	2.89	3.28	2.75	3.67
328.15	3.63	3.92	2.83	3.20	2.68	3.59
338.15	3.53	3.81	2.76	3.12	2.62	3.51
348.15	3.44	3.71	2.71	3.06	2.57	3.44
358.15	3.36	3.63	2.65	3.00	2.52	3.38
***T*****/K**	**1-octene**	**1-decene**	**1-hexyne**	**1-heptyne**	**1-octyne**	**benzene**
318.15	4.09	4.91	2.10	2.48	2.89	0.886
328.15	4.00	4.80	2.08	2.45	2.84	0.887
338.15	3.91	4.70	2.05	2.41	2.79	0.888
348.15	3.83	4.61	2.03	2.39	2.76	0.888
358.15	3.75	4.51	2.01	2.35	2.71	0.888
***T*****/K**	**toluene**	**ethylbenzene**	***o*****-xylene**	***m*****-xylene**	***p*****-xylene**	**methanol**
318.15	1.18	1.62	1.38	1.51	1.51	0.896
328.15	1.18	1.61	1.38	1.51	1.51	0.850
338.15	1.18	1.60	1.38	1.51	1.51	0.805
348.15	1.18	1.58	1.38	1.51	1.51	0.761
358.15	1.18	1.58	1.38	1.51	1.51	0.719
***T*****/K**	**ethanol**	**1-propanol**	**1-butanol**	**water**	**acetic acid**	**thiophene**
318.15	0.885	1.02	1.23	2.21	−0.614	0.754
328.15	0.836	0.968	1.17	2.13	−0.534	0.756
338.15	0.788	0.918	1.11	2.06	−0.464	0.756
348.15	0.745	0.868	1.05	1.99	−0.397	0.757
358.15	0.698	0.822	0.995	1.94	−0.334	0.756
***T*****/K**	**tetrahydrofuran**	**1,4-dioxane**	**methyl*****tert-*****butyl ether**	**methyl*****tert-*****pentyl ether**	**diethyl ether**	**di-*****n*****-propyl ether**
318.15	0.125	−0.205	1.26	1.69	1.34	2.43
328.15	0.166	−0.157	1.29	1.70	1.35	2.40
338.15	0.201	−0.112	1.31	1.71	1.36	2.38
348.15	0.230	−0.070	1.33	1.73	1.37	2.36
358.15	0.260	−0.032	1.35	1.74	1.38	2.35
***T*****/K**	**di-*****n*****-butyl ether**	**acetone**	**2-pentanone**	**3-pentanone**		
318.15	3.30	−0.351	0.193	0.253		
328.15	3.25	−0.314	0.217	0.277		
338.15	3.20	−0.284	0.242	0.301		
348.15	3.16	−0.255	0.261	0.322		
358.15	3.12	−0.229	0.281	0.341		

**[emim][TCB]**						

***T*****/K**	***n*****-pentane**	***n*****-hexane**	***n*****-heptane**	***n*****-octane**	**2,2,4-trimethylpentane**	***n*****-nonane**
298.15	3.26	3.63	4.05	4.46	4.14	4.90
308.15	3.16	3.54	3.94	4.34	4.03	4.76
318.15	3.11	3.47	3.86	4.25	3.96	4.65
328.15	3.02	3.38	3.75	4.14	3.87	4.52
338.15	2.96	3.30	3.67	4.04	3.78	4.41
348.15	2.90	3.24	3.60	3.95	3.71	4.31
358.15	2.84	3.18	3.52	3.86	3.63	4.21
***T*****/K**	***n*****-decane**	**cyclopentane**	**cyclohexane**	**methylcyclohe xane**	**cycloheptane**	**cyclooctane**
298.15	5.32	2.64	3.01	3.35	3.23	3.49
308.15	5.18	2.57	2.92	3.24	3.13	3.38
318.15	5.06	2.52	2.86	3.17	3.07	3.31
328.15	4.92	2.44	2.77	3.08	2.98	3.21
338.15	4.81	2.39	2.70	3.00	2.90	3.14
348.15	4.69	2.34	2.64	2.94	2.83	3.06
358.15	4.58	2.28	2.57	2.88	2.77	2.98
***T*****/K**	**1-pentene**	**1-hexene**	**cyclohexene**	**1-heptene**	**1-octene**	**1-hexyne**
298.15	2.42	2.80	2.21	3.18	3.61	1.50
308.15	2.37	2.73	2.16	3.10	3.52	1.49
318.15	2.34	2.69	2.14	3.06	3.46	1.49
328.15	2.29	2.62	2.09	2.99	3.37	1.48
338.15	2.25	2.57	2.04	2.92	3.30	1.48
348.15	2.22	2.53	2.01	2.89	3.24	1.47
358.15	2.15	2.48	1.98	2.83	3.18	1.47
***T*****/K**	**1-heptyne**	**1-octyne**	**benzene**	**toluene**	**ethylbenzene**	***o*****-xylene**
298.15	1.86	2.23	0.433	0.710	1.10	0.922
308.15	1.84	2.21	0.443	0.721	1.10	0.927
318.15	1.83	2.18	0.455	0.730	1.10	0.933
328.15	1.81	2.16	0.462	0.739	1.10	0.937
338.15	1.80	2.14	0.471	0.747	1.10	0.943
348.15	1.78	2.12	0.477	0.757	1.09	0.949
358.15	1.77	2.11	0.483	0.762	1.09	0.950
***T*****/K**	***m*****-xylene**	***p*****-xylene**	**methanol**	**ethanol**	**1-propanol**	**1-butanol**
298.15	1.08	1.02	0.968	1.04	1.11	1.29
308.15	1.08	1.03	0.886	0.944	1.01	1.17
318.15	1.09	1.03	0.812	0.856	0.909	1.06
328.15	1.09	1.04	0.739	0.770	0.816	0.953
338.15	1.10	1.05	0.674	0.693	0.734	0.861
348.15	1.10	1.06	0.612	0.620	0.660	0.780
358.15	1.10	1.06	0.553	0.551	0.591	0.701
***T*****/K**	**water**	**thiophene**	**tetrahydrofuran**	**methyl*****tert-*****butyl ether**	**methyl*****tert-*****pentyl ether**	**diethyl ether**
298.15	2.39	0.316	−0.0164	1.19	1.53	1.21
308.15	2.27	0.325	0.0104	1.20	1.54	1.21
318.15	2.19	0.331	0.0335	1.21	1.54	1.21
328.15	2.10	0.337	0.0458	1.22	1.55	1.21
338.15	2.01	0.345	0.0626	1.23	1.55	1.21
348.15	1.92	0.348	0.0878	1.24	1.56	1.21
358.15	1.85	0.355	0.101	1.24	1.56	1.20
***T*****/K**	**di-*****n*****-propyl ether**	**di-*****n*****-butyl ether**	**acetone**	**2-pentanone**	**3-pentanone**	**2-hexanone**
298.15	2.24	3.06	−0.445	−0.0425	−0.0790	0.210
308.15	2.21	2.99	−0.421	−0.0239	−0.0528	0.225
318.15	2.18	2.94	−0.398	−0.0018	−0.0208	0.238
328.15	2.14	2.87	−0.379	0.0155	0.0047	0.247
338.15	2.12	2.83	−0.358	0.0298	0.0266	0.261
348.15	2.09	2.78	−0.344	0.0427	0.0464	0.272
358.15	2.06	2.73	−0.325	0.0601	0.0678	0.283
***T*****/K**	**3-hexanone**					
298.15	0.276					
308.15	0.294					
318.15	0.314					
328.15	0.330					
338.15	0.343					
348.15	0.355					
358.15	0.371					

**[dmim][TCB]**						

***T*****/K**	***n*****-pentane**	***n*****-hexane**	***n*****-heptane**	***n*****-octane**	**2,2,4-trimethylpentane**	***n*****-nonane**
328.15	1.98	2.11	2.27	2.44	2.35	2.62
338.15	1.94	2.07	2.23	2.39	2.30	2.57
348.15	1.90	2.03	2.18	2.34	2.25	2.52
358.15	1.85	1.99	2.13	2.30	2.21	2.47
368.15	1.81	1.94	2.09	2.25	2.17	2.42
***T*****/K**	***n*****-decane**	**cyclopentane**	**cyclohexane**	**methylcyclohe xane**	**cycloheptane**	**cyclooctane**
328.15	2.82	1.58	1.73	1.84	1.78	1.88
338.15	2.76	1.54	1.68	1.79	1.73	1.83
348.15	2.71	1.50	1.63	1.75	1.69	1.79
358.15	2.65	1.46	1.59	1.71	1.65	1.74
368.15	2.60	1.42	1.54	1.67	1.61	1.70
***T*****/K**	**1-pentene**	**1-hexene**	**cyclohexene**	**1-heptene**	**1-octene**	**1-hexyne**
328.15	1.49	1.63	1.28	1.78	1.96	0.853
338.15	1.47	1.59	1.25	1.75	1.93	0.857
348.15	1.45	1.56	1.23	1.73	1.90	0.860
358.15	1.42	1.53	1.21	1.70	1.87	0.859
368.15	1.40	1.51	1.18	1.68	1.84	0.861
***T*****/K**	**1-heptyne**	**1-octyne**	**benzene**	**toluene**	**ethylbenzene**	***o*****-xylene**
328.15	0.983	1.14	0.0698	0.182	0.382	0.266
338.15	0.987	1.14	0.0826	0.201	0.396	0.283
348.15	0.990	1.14	0.0957	0.218	0.409	0.302
358.15	0.991	1.14	0.105	0.233	0.421	0.318
368.15	0.992	1.14	0.114	0.247	0.429	0.330
***T*****/K**	***m*****-xylene**	***p*****-xylene**	**methanol**	**ethanol**	**1-propanol**	**1-butanol**
328.15	0.361	0.343	0.997	0.835	0.682	0.635
338.15	0.381	0.366	0.929	0.759	0.613	0.565
348.15	0.402	0.386	0.870	0.693	0.555	0.507
358.15	0.416	0.401	0.803	0.625	0.497	0.452
368.15	0.437	0.422	0.752	0.566	0.441	0.392
***T*****/K**	**water**	**acetic acid**	**butyric acid**	**thiophene**	**tetrahydrofuran**	**methyl*****tert-*****butyl ether**
328.15	2.78	−0.332	0.118	0.0626	−0.338	0.547
338.15	2.68	−0.284	0.116	0.0761	−0.307	0.565
348.15	2.57	−0.238	0.114	0.0845	−0.279	0.585
358.15	2.49	−0.198	0.111	0.0968	−0.255	0.605
368.15	2.42	−0.164	0.109	0.107	−0.230	0.619
***T*****/K**	**methyl*****tert-*****pentyl ether**	**diethyl ether**	**di-*****n*****-propyl ether**	**di-*****n*****-butyl ether**	**acetone**	**2-pentanone**
328.15	0.711	0.626	1.15	1.49	−0.450	−0.462
338.15	0.728	0.635	1.14	1.48	−0.428	−0.431
348.15	0.746	0.641	1.13	1.47	−0.409	−0.404
358.15	0.760	0.646	1.13	1.46	−0.394	−0.380
368.15	0.772	0.649	1.12	1.45	−0.378	−0.353
***T*****/K**	**3-pentanone**					
328.15	−0.497					
338.15	−0.460					
348.15	−0.426					
358.15	−0.395					
368.15	−0.364					

**[bmPIP][SCN]**						

***T*****/K**	***n*****-hexane**	***n*****-heptane**	***n*****-octane**	***n*****-nonane**	***n*****-decane**	**cyclopentane**
318.15	4.90	5.19	5.49	5.82	6.19	3.55
328.15	4.73	5.00	5.36	5.69	6.07	3.42
338.15	4.57	4.89	5.24	5.60	5.97	3.32
348.15	4.44	4.75	5.11	5.46	5.84	3.21
358.15	4.30	4.67	5.03	5.37	5.74	3.15
***T*****/K**	**cyclohexane**	**cycloheptane**	**cyclooctane**	**1-hexene**	**1-heptene**	**1-octene**
318.15	3.85	3.91	4.17	3.82	4.16	4.54
328.15	3.74	3.84	4.07	3.71	4.08	4.46
338.15	3.64	3.74	3.97	3.64	4.00	4.38
348.15	3.54	3.65	3.88	3.56	3.92	4.30
358.15	3.46	3.60	3.81	3.49	3.87	4.24
***T*****/K**	**1-hexyne**	**1-heptyne**	**1-octyne**	**benzene**	**toluene**	**ethylbenzene**
318.15	1.94	2.30	2.66	0.907	1.32	1.76
328.15	1.94	2.30	2.66	0.916	1.33	1.76
338.15	1.95	2.30	2.66	0.924	1.33	1.75
348.15	1.95	2.30	2.66	0.930	1.33	1.75
358.15	1.95	2.30	2.66	0.938	1.34	1.74
***T*****/K**	***o*****-xylene**	***m*****-xylene**	***p*****-xylene**	**methanol**	**ethanol**	**water**
318.15	1.54	1.77	1.72	−0.187	0.103	0.413
328.15	1.55	1.77	1.73	−0.190	0.0822	0.429
338.15	1.55	1.77	1.73	−0.191	0.0600	0.445
348.15	1.56	1.77	1.73	−0.196	0.0405	0.460
358.15	1.56	1.77	1.74	−0.198	0.0241	0.476
***T*****/K**	**thiophene**	**tetrahydrofuran**	**methyl*****tert-*****butyl ether**	**diethyl ether**	**di-*****n*****-propyl ether**	**di-*****n*****-butyl ether**
318.15	0.434	1.14	2.70	2.67	3.63	4.39
328.15	0.459	1.15	2.66	2.62	3.56	4.31
338.15	0.486	1.16	2.63	2.57	3.50	4.24
348.15	0.504	1.16	2.59	2.54	3.44	4.17
358.15	0.525	1.17	2.57	2.50	3.39	4.12
***T*****/K**	**acetone**	**2-pentanone**	**3-pentanone**			
318.15	0.795	1.29	1.30			
328.15	0.794	1.29	1.30			
338.15	0.792	1.29	1.30			
348.15	0.790	1.29	1.30			
358.15	0.789	1.29	1.30			

**[pmPIP][NTf****_2_****]**						

***T*****/K**	***n*****-pentane**	***n*****-hexane**	***n*****-heptane**	***n*****-octane**	***n*****-nonane**	***n*****-decane**
308.15	3.31	3.40	3.58	3.81	4.08	4.38
318.15	3.02	3.21	3.44	3.70	3.98	4.30
328.15	2.98	3.14	3.35	3.60	3.88	4.18
338.15	2.85	3.06	3.29	3.53	3.80	4.09
348.15	2.75	2.95	3.19	3.43	3.70	3.99
358.15	2.72	2.93	3.14	3.37	3.63	3.91
***T*****/K**	**cyclopentane**	**cyclohexane**	**cycloheptane**	**cyclooctane**	**1-pentene**	**1-hexene**
308.15	2.72	2.93	3.05	3.23	2.48	2.64
318.15	2.52	2.77	2.94	3.15	2.30	2.52
328.15	2.47	2.71	2.86	3.04	2.27	2.48
338.15	2.38	2.62	2.78	2.98	2.32	2.41
348.15	2.29	2.51	2.70	2.89	2.13	2.34
358.15	2.26	2.48	2.64	2.82	2.13	2.31
***T*****/K**	**1-heptene**	**1-octene**	**1-hexyne**	**1-heptyne**	**1-octyne**	**benzene**
308.15	2.84	3.11	1.47	1.71	1.98	0.418
318.15	2.76	3.04	1.46	1.70	1.98	0.427
328.15	2.71	2.96	1.45	1.68	1.95	0.430
338.15	2.65	2.92	1.46	1.69	1.94	0.433
348.15	2.59	2.85	1.44	1.67	1.95	0.444
358.15	2.55	2.81	1.44	1.67	1.91	0.456
***T*****/K**	**toluene**	**ethylbenzene**	***o*****-xylene**	***m*****-xylene**	***p*****-xylene**	**methanol**
308.15	0.615	0.913	0.752	0.822	0.812	1.62
318.15	0.619	0.915	0.772	0.851	0.845	1.54
328.15	0.635	0.932	0.775	0.849	0.849	1.44
338.15	0.642	0.930	0.787	0.865	0.864	1.36
348.15	0.655	0.936	0.795	0.875	0.876	1.28
358.15	0.668	0.942	0.811	0.892	0.894	1.20
***T*****/K**	**ethanol**	**1-propanol**	**1-butanol**	**water**	**thiophene**	**tetrahydrofuran**
308.15	1.54	1.55	1.64	3.36	0.377	0.355
318.15	1.45	1.46	1.56	3.27	0.386	0.363
328.15	1.35	1.35	1.42	3.14	0.389	0.370
338.15	1.26	1.26	1.32	3.04	0.401	0.385
348.15	1.18	1.17	1.23	2.90	0.404	0.384
358.15	1.10	1.10	1.15	2.78	0.416	0.398
***T*****/K**	**methyl*****tert-*****butyl ether**	**diethyl ether**	**di-*****n*****-propyl ether**	**di-*****n*****-butyl ether**	**acetone**	**2-pentanone**
308.15	1.38	1.47	2.16	2.78	−0.0329	0.181
318.15	1.37	1.41	2.12	2.72	−0.0217	0.200
328.15	1.36	1.41	2.10	2.68	−0.0151	0.209
338.15	1.35	1.39	2.07	2.63	0.0007	0.225
348.15	1.34	1.36	2.02	2.57	−0.0029	0.229
358.15	1.33	1.36	2.01	2.53	0.0071	0.244
***T*****/K**	**3-pentanone**					
308.15	0.161					
318.15	0.178					
328.15	0.208					
338.15	0.225					
348.15	0.235					
358.15	0.256					

**[bmPIP][NTf****_2_****]**						

***T*****/K**	***n*****-pentane**	***n*****-hexane**	**3-methylpentane**	**2,2-dimethylbutane**	***n*****-heptane**	***n*****-octane**
308.15	2.62	2.85	2.74	2.66	3.13	3.42
318.15	2.51	2.72	2.62	2.54	3.00	3.27
328.15	2.39	2.62	2.50	2.42	2.89	3.16
338.15	2.34	2.56	2.45	2.37	2.82	3.09
348.15	2.29	2.51	2.40	2.33	2.76	3.02
358.15	2.23	2.47	2.35	2.28	2.69	2.95
***T*****/K**	**2,2,4-trimethylpentane**	***n*****-nonane**	***n*****-decane**	**cyclopentane**	**cyclohexane**	**methylcyclohe xane**
308.15	3.03	3.71	4.01	2.22	2.48	2.67
318.15	2.92	3.55	3.84	2.11	2.36	2.54
328.15	2.81	3.42	3.71	2.02	2.26	2.44
338.15	2.74	3.35	3.63	1.96	2.20	2.38
348.15	2.69	3.28	3.54	1.90	2.12	2.31
358.15	2.63	3.20	3.47	1.87	2.09	2.27
***T*****/K**	**cycloheptane**	**cyclooctane**	**1-pentene**	**1-hexene**	**cyclohexene**	**1-heptene**
308.15	2.70	2.91	2.00	2.26	1.95	2.51
318.15	2.57	2.78	1.91	2.16	1.87	2.41
328.15	2.47	2.67	1.83	2.08	1.79	2.33
338.15	2.40	2.60	1.81	2.03	1.75	2.29
348.15	2.32	2.51	1.76	1.97	1.70	2.23
358.15	2.28	2.47	1.73	1.94	1.64	2.21
***T*****/K**	**1-octene**	**1-hexyne**	**1-heptyne**	**1-octyne**	**benzene**	**toluene**
308.15	2.80	1.28	1.52	1.79	0.304	0.486
318.15	2.70	1.24	1.47	1.72	0.315	0.498
328.15	2.60	1.20	1.43	1.67	0.317	0.508
338.15	2.55	1.20	1.42	1.66	0.324	0.518
348.15	2.49	1.18	1.40	1.65	0.338	0.534
358.15	2.46	1.20	1.40	1.63	0.354	0.550
***T*****/K**	**ethylbenzene**	***o*****-xylene**	***m*****-xylene**	***p*****-xylene**	**methanol**	**ethanol**
308.15	0.793	0.632	0.702	0.698	1.60	1.49
318.15	0.800	0.646	0.723	0.716	1.52	1.40
328.15	0.795	0.644	0.718	0.719	1.42	1.31
338.15	0.797	0.650	0.729	0.733	1.34	1.21
348.15	0.807	0.667	0.748	0.747	1.25	1.12
358.15	0.816	0.671	0.759	0.758	1.16	1.04
***T*****/K**	**1-propanol**	**1-butanol**	**water**	**thiophene**	**tetrahydrofuran**	**methyl*****tert-*****butyl ether**
308.15	1.47	1.54	3.49	0.299	0.206	1.06
318.15	1.38	1.42	3.34	0.302	0.215	1.06
328.15	1.28	1.33	3.21	0.301	0.205	1.05
338.15	1.18	1.22	3.07	0.306	0.207	1.04
348.15	1.09	1.12	2.94	0.308	0.207	1.04
358.15	1.01	1.04	2.81	0.323	0.219	1.04
***T*****/K**	**diethyl ether**	**di-*****n*****-propyl ether**	**di-*****n*****-butyl ether**	**acetone**	**2-pentanone**	**3-pentanone**
308.15	1.19	1.86	2.46	−0.0841	0.0558	0.0284
318.15	1.18	1.85	2.43	−0.0782	0.0667	0.0492
328.15	1.11	1.75	2.33	−0.0764	0.0885	0.0770
338.15	1.12	1.75	2.29	−0.0739	0.0943	0.0842
348.15	1.11	1.73	2.25	−0.0732	0.106	0.114
358.15	1.10	1.70	2.21	−0.0706	0.115	0.124

**[OQuin][NTf****_2_****]**						

***T*****/K**	***n*****-pentane**	***n*****-hexane**	***n*****-heptane**	***n*****-octane**	***n*****-nonane**	***n*****-decane**
328.15	1.90	2.00	2.17	2.30	2.46	2.65
338.15	1.86	1.97	2.12	2.26	2.42	2.60
348.15	1.82	1.93	2.07	2.21	2.37	2.54
358.15	1.79	1.90	2.03	2.18	2.33	2.50
368.15	1.75	1.87	1.99	2.14	2.29	2.45
***T*****/K**	**cyclopentane**	**cyclohexane**	**cycloheptane**	**cyclooctane**	**1-pentene**	**1-hexene**
328.15	1.57	1.70	1.76	1.85	1.50	1.60
338.15	1.54	1.66	1.72	1.81	1.48	1.58
348.15	1.50	1.61	1.68	1.76	1.46	1.55
358.15	1.47	1.58	1.64	1.72	1.43	1.53
368.15	1.43	1.54	1.61	1.68	1.41	1.51
***T*****/K**	**1-heptene**	**1-octene**	**1-hexyne**	**1-heptyne**	**1-octyne**	**benzene**
328.15	1.74	1.89	0.977	1.09	1.22	0.188
338.15	1.72	1.87	0.982	1.09	1.22	0.194
348.15	1.70	1.84	0.981	1.09	1.21	0.206
358.15	1.68	1.82	0.983	1.09	1.22	0.216
368.15	1.66	1.80	0.987	1.08	1.22	0.224
***T*****/K**	**toluene**	**ethylbenzene**	***o*****-xylene**	***m*****-xylene**	***p*****-xylene**	**methanol**
328.15	0.247	0.471	0.255	0.386	0.399	1.62
338.15	0.267	0.485	0.282	0.402	0.416	1.54
348.15	0.285	0.495	0.303	0.419	0.424	1.43
358.15	0.302	0.505	0.322	0.430	0.437	1.35
368.15	0.320	0.517	0.340	0.445	0.447	1.27
***T*****/K**	**ethanol**	**1-propanol**	**1-butanol**	**1-pentanol**	**water**	**thiophene**
328.15	1.43	1.29	1.24	1.15	3.53	0.215
338.15	1.32	1.20	1.14	1.07	3.40	0.223
348.15	1.22	1.10	1.02	0.979	3.24	0.227
358.15	1.14	1.02	0.943	0.896	3.12	0.231
368.15	1.06	0.942	0.865	0.827	2.98	0.236
***T*****/K**	**tetrahydrofuran**	**methyl*****tert-*****butyl ether**	**diethyl ether**	**di-*****n*****-propyl ether**	**di-*****n*****-butyl ether**	**acetone**
328.15	0.0326	0.778	0.879	1.33	1.65	−0.0485
338.15	0.0506	0.787	0.880	1.31	1.63	−0.0465
348.15	0.0653	0.793	0.881	1.30	1.60	−0.0456
358.15	0.0742	0.796	0.879	1.28	1.57	−0.0458
368.15	0.0853	0.803	0.877	1.26	1.54	−0.0465
***T*****/K**	**2-pentanone**	**3-pentanone**				
328.15	−0.0778	−0.0596				
338.15	−0.0694	−0.0483				
348.15	−0.0621	−0.0372				
358.15	−0.0521	−0.0285				
368.15	−0.0461	−0.0216				

### Example of Calculation of the Solubility Parameter

Experimental data for *n*-octane + [emim][TCB] system at *T* = 298.15 K:

*T* = 298.15 K
*p*_i_ = 137423 Pa
*p*_o_ = 97423 Pa
*T**_f_* = 297.15 K
*U* = 41.2 mL·min^−1^
*t*_R_ − *t*_G_ = 270.66 s
*m*_2_ = 2.1053 g
*P*_w_ (at *T**_f_*) = 2986.2 Pa (from [30])
*U*_o_ = 6.679·10^−7^ m^3^·s^−1^ (from Equation 6)
*J*_2_^3^ = 1.217 (from Equation 5)
*V*_N_ = 1.485·10^−4^ m^3^ (from Equation 4)
*V**_g_* = 6.464·10^−5^ m^3^·g^−1^ (from Equation 3)
*P*_1_^*^ = 1871.0 Pa (from [30])
*M*_1_ = 114.2285 g·mol^−1^ (from [30])
*B*_11_ = −4.496·10^−3^ m^3^·mol^−1^ (from [30])
*V*_1_^*^ = 1.6256·10^−4^ m^3^·mol^−1^ (from [30])
*V*_2_^*^ = 2.1818·10^−4^ m^3^·mol^−1^ (calculated from density from [19])
*ρ*_1_ = 0.70268 g·cm^−3^ (from [30])
*ρ*_2_ = 1.03627 g·cm^−3^ (from [19])
*χ*_12_^∞^ = 4.463 (from Equation 2)
*δ*_1_ = 15486 (J·m^3^)_0.5_ (from [30])

Analogous calculations were made for the rest of solutes. The results are presented in the Table 1S. Based on these values the Equation 7 can be plotted (see Figure S1).

From the slope (2*δ*_2_/R*T*) the value of 20.874 is obtained. From this value the *δ*_2_ is calculated giving value 25.9 MPa^0.5^ (see Table 2).

### Calculation of the *K*_v_ Constant from Equation 2

Using data presented in the Table S2 and the *K*_v_ value of 7.8 the solubility parameters were determined using Equation 2. Then the *K*_v_ value was optimized using the objective function $\text{OF}=\sum_{i=1}^{n}{\left(\delta_{\text{experimental}}-\delta_{\text{calculated}}\right)}_{i}^{2}$ using MS Excel Solver. Densities and viscosities were taken from the ILThermo database available at http://ilthermo.boulder.nist.gov/ILThermo/. Solubility parameters were calculated from enthalpies of vaporization [25–29].

**Table S2:** Data used in calculation of *K*_v_ constant.

Ionic Liquid	*ρ*/g·cm^−3^	*M*/g·mol^−1^	*μ*/mPa·s	*υ*/cm^3^·mol^−1^	*δ*_2_/MPa^0.5^
[emim][NTf_2_]	1.5192	391.32	34.29	257.6	21.3
	1.5192	391.32	34.29	257.6	22.6
	1.5192	391.32	34.29	257.6	22.7

[bmim][NTf_2_]	1.4366	419.37	50.70	291.9	21.2
	1.4366	419.37	50.70	291.9	19.8
	1.4366	419.37	50.70	291.9	22.9

[hmim][NTf_2_]	1.3706	447.42	70.96	326.5	20.5
	1.3706	447.42	70.96	326.5	19.0
	1.3706	447.42	70.96	326.5	22.9

[omim][NTf_2_]	1.3206	475.48	92.51	360.1	20.2
	1.3206	475.48	92.51	360.1	20.2
	1.3206	475.48	92.51	360.1	23.0
	1.3206	475.48	92.51	360.1	18.9

[dmim][NTf_2_]	1.2780	499.50	108.20	390.8	17.8

[bmPYR][NTf_2_]	1.3940	422.41	76.92	303.0	22.2

**Table S3:** Densities and viscosities for [*N*-C_3_OHPY][NTf_2_], [pmPIP][NTf_2_] and [bmPIP][NTf_2_] ionic liquids.

Ionic Liquid	*T*/K	*ρ*/g·cm^−3^^a^	*μ*/m Pa·s b
[*N*-C_3_OHPY][NTf_2_]	308.15	1.5451	67.03
	318.15	1.5357	43.85
	328.15	1.5266	30.56
	338.15	1.5175	22.21
	348.15	1.5085	16.90

[pmPIP][NTf_2_]	308.15	1.4010	86.70
	318.15	1.3923	55.24
	328.15	1.3837	37.75
	338.15	1.3751	27.17
	348.15	1.3666	20.32

[bmPIP][NTf_2_]	308.15	1.3706	97.76
	318.15	1.3621	61.05
	328.15	1.3536	40.92
	338.15	1.3452	29.01
	348.15	1.3369	21.28

a determined using Anton Paar DMA 4500 densitometer;

b determined using Anton Paar AMVn viscometer.
